# Predictive value and clinical guidance of FNA-Tg combined with BRAF V600E for PTC cervical lymph node metastasis: a case report

**DOI:** 10.3389/fsurg.2026.1837054

**Published:** 2026-07-16

**Authors:** Wen-shi Yang, Feng-feng Zhang, Ming-rui Song, Yun Li

**Affiliations:** 1 Department of Breast and Thyroid Surgery, The Affiliated Tengzhou Central People’s Hospital of Xuzhou Medical University, Tengzhou, China; 2 Department of the Pathology, The Affiliated Tengzhou Central People’s Hospital of Xuzhou Medical University, Tengzhou, China; 3 Department of Operating Room, The Affiliated Tengzhou Central People’s Hospital of Xuzhou Medical University, Tengzhou, China

**Keywords:** BRAF v600E, cervical lymph node metastasis, FNA-Tg, papillary thyroid carcinoma, thyroid cancer

## Abstract

This study reports a rare case in which the integration of Thyroglobulin measurement in the needle washout after fine-needle aspiration (FNA-Tg) and BRAF V600E analysis rectified a histopathological misdiagnosis of cervical lymph node metastasis originating from papillary thyroid carcinoma. The patient was a 52-year-old female farmer who presented with a diagnosis of papillary thyroid carcinoma. Histopathological assessment of cervical lymph nodes returned negative findings, whereas both FNA-Tg and BRAF V600E are positive results. Following a comprehensive re-evaluation of the histopathological specimens, a definitive diagnosis of a rare case of cervical lymph node micrometastasis was confirmed. This case report provides a systematic review of the current literature and critically examines the diagnostic utility of combining FNA-Tg with BRAF V600E in the detection of cervical lymph node metastases from PTC, potentially offering a therapeutic strategy for the clinical management of such conditions.

## Introduction

PTC is the most frequent histological subtype of thyroid malignancy, accounting for 80% to 90% of all thyroid carcinomas (1). Pathological examination reveals that approximately 30% to 80% of patients with PTC exhibit LNM, which is one of the important risk factors for local recurrence, distant metastasis, and low survival rates (2). Therefore, accurately assessing cervical LNM in PTC patients is crucial for individualized therapy, as it enables both the avoidance of overdiagnosis and the prevention of under-treatment. Ultrasound-guided fine-needle aspiration cytology (FNAC) is a standard diagnostic modality by the guidelines to detect early thyroid carcinoma and suspicious lymph node metastasis (LNM), including patients with PTC undergoing follow-up after total thyroidectomy (3). However, due to the limited number of cells obtained from fine-needle aspiration of lymph nodes, the clinical misdiagnosis rate reaches 20% to 30%. In addition, the results can also be influenced by the level of the clinician, which limits its widespread clinical application (4). Tg is a specific glycoprotein produced only by PTC cells or normal thyroid follicular cells. Therefore, FNA-Tg can serve as an excellent complement to FNAC (5). However, the optimal cutoff value and whether it is affected by serum Tg levels remain controversial (6). BRAF V600E mutation has been thought to be a valuable molecular marker for the diagnosis of PTC. However, whether the BRAF V600E mutation is associated with LNM remains controversial (7). The patient in the present case is a 52-year-old female farmer with PTC, whose histopathological diagnosis of cervical LNM is misjudged, whereas both FNA-Tg and BRAF V600E are positive results. After surgery, the patient received more intensive TSH suppression therapy. Follow-up assessments conducted at 6 months, no significant signs of recurrence were observed. This case report suggests that BRAF V600E mutation measurement in the needle washout after fine-needle aspiration is a feasible approach for diagnosing suspected cervical LNM in patients with PTC. Combination of FNA-Tg and BRAF V600E enables both avoidance of overdiagnosis and prevention of under-treatment, while offering new clinical perspectives for the clinical management of this condition.

## Case report

A 52-year-old female patient underwent an ultrasound examination at The Affiliated Tengzhou City Central People's Hospital of Xuzhou Medical University on December 01, 2024, and a thyroid mass classified as TI-RADS 4B was discovered. The patient has a 1-year history of hypertension with well-controlled blood pressure. She denies any family history of genetic diseases or a history of radiation exposure. After physical examination and FNAC, she was diagnosed with PTC and was subsequently admitted to our hospital for surgical treatment.

Ultrasound examination results showed that a nodule measuring approximately 0.26 × 0.33 cm was detected on the ventral side of the left lobe of the thyroid. It had an unclear margin and an irregular shape, with an aspect ratio greater than 1.

**Figure 1:**
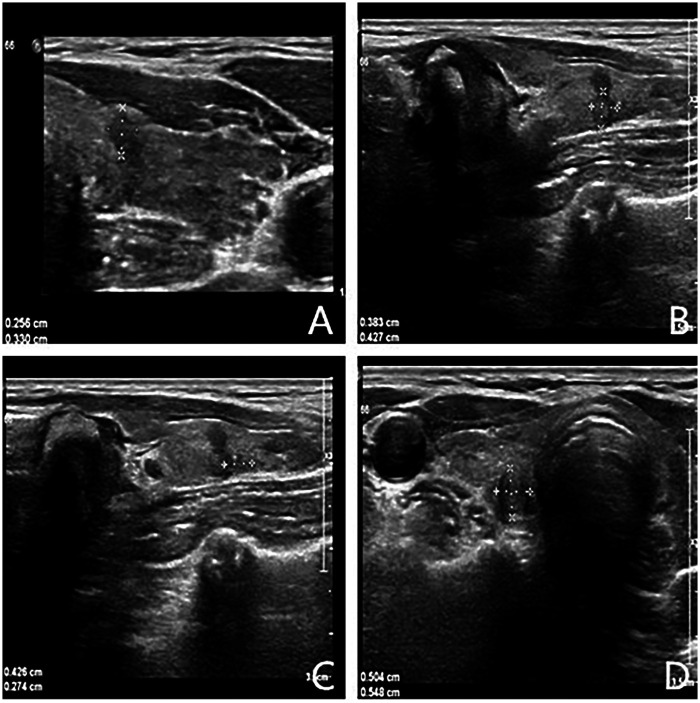
(**A**) Nodule in the ventral aspect of the left lobe of thyroid (**B**) Nodule 1 in the dorsal aspect of the left lobe of thyroid (**C**) Nodule 2 in the dorsal aspect of the left lobe of thyroid (**D**) Nodule in the right lobe of thyroid.

Additionally, nodules of approximately 0.38 × 0.43 cm (aspect ratio greater than 1) and 0.43 × 0.27 cm were detected on the dorsal side of the left lobe. Both have unclear margins and irregular shapes, with punctate strong echoes visible inside. The nodules are classified as TI-RADS 4B (Figures 1A–D). No significant abnormal lymphadenopathy was detected on neck ultrasonography.

The laboratory tests, electrocardiogram (ECG), and Physical examination no significant abnormalities. She was informed of the efficacy, risks, and alternative options for surgical treatment. The patient agreed to the treatment plan and signed the surgical consent form.

## Surgical technique

On December 4, 2024, the patient underwent total thyroidectomy and central neck dissection under general anesthesia. Intraoperative exploration revealed multiple masses in both thyroid lobes. The largest mass, located on the right side, measured approximately 0.6 cm × 0.5 cm × 0.4 cm, with firm consistency and ill-defined borders. During concurrent prophylactic central neck lymph node dissection, one cystic lymph node with a diameter of approximately 0.6 cm was detected intraoperatively and clinically suspected to be a metastatic lymph node. Therefore, this lymph node was labeled as A for intra-operative direct-vision fine-needle aspiration biopsy.

To facilitate the procurement of high-quality pathological specimens, a 10 mL disposable syringe coupled to a 23-gauge needle was employed in this investigation. Under direct intraoperative visualization, the fine needle was inserted into the target lymph node, and reciprocal aspiration was conducted within the lymph node for a minimum of two cycles under negative pressure to harvest cellular material. Notably, complete evacuation of cystic fluid is not mandatory for cystic lymph node lesions. Upon the release of negative pressure, the needle was withdrawn promptly, and the aspirated cellular specimen in the needle lumen was repeatedly washed into an EP tube pre-filled with 0.5 mL of physiological saline. The thyroglobulin (Tg) concentration of the harvested specimens was quantified using electrochemiluminescence immunoassay (ECLIA). Concurrently, an aliquot of the aspirated specimen was repeatedly washed into an EP tube containing 0.5 mL of cell preservation solution, and detection of BRAF V600E gene mutation status was performed in strict adherence to the manufacturer's protocol provided with the commercial human BRAF V600E mutation detection kit. The punctured lymph node was marked as specimen A and submitted for histopathological examination, with histopathological diagnosis employed as the gold standard to identify the presence of cervical LNM.

## Diagnostic examination

**Figure 2:**
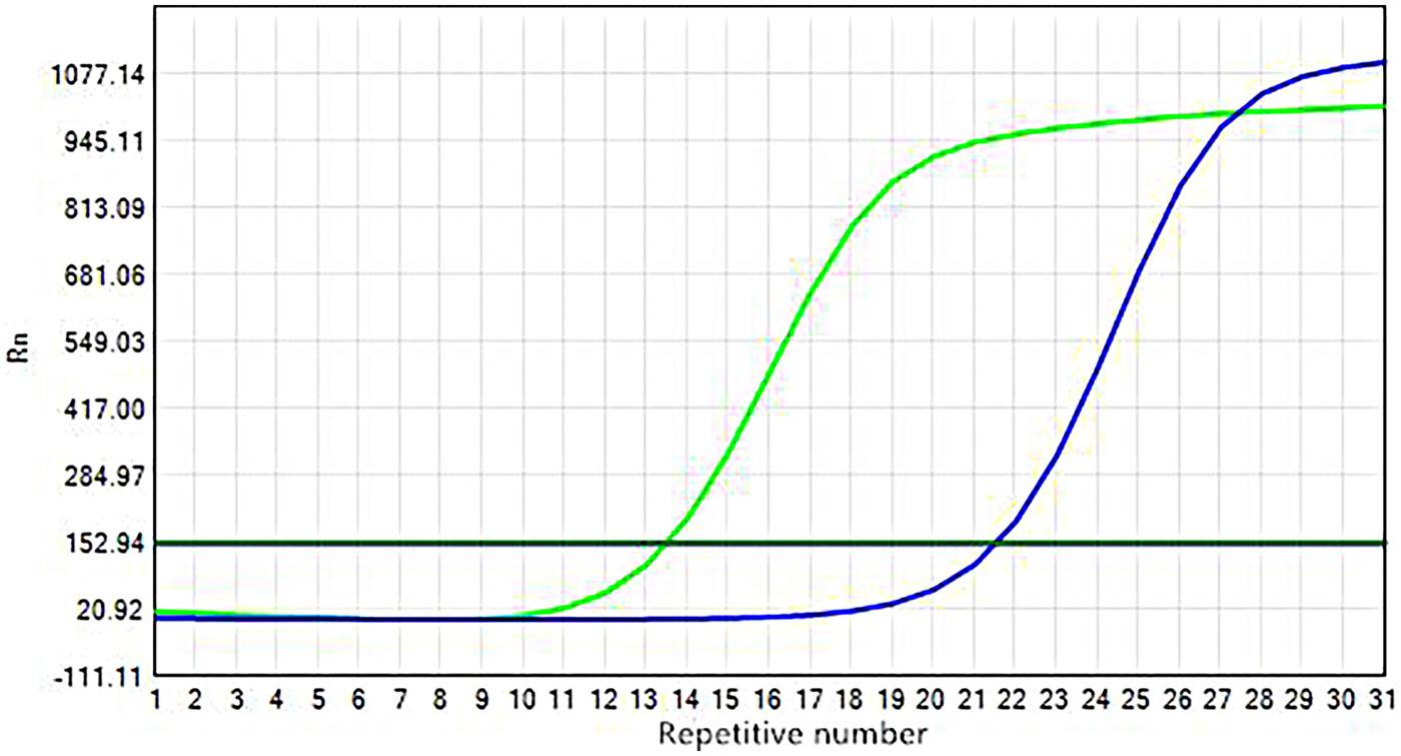
Fluorescence quantitative PCR amplification curve plot for BRAF V600E gene detection in lymph nodes A (Amplification curve of internal reference gene; Amplification curve of BRAF V600E).

FNA-Tg result was > 500 ng/mL; Experimental data demonstrate that the amplification curve of the BRAF V600E mutation exhibits a typical “S”-shaped pattern, indicating the presence of the V600E mutation in exon 15 of the BRAF gene within the sample (Figure 2). The amplification curve of the internal reference gene is normal, confirming the accuracy and reliability of the results. Which suggesting that lymph node A is a metastatic lesion of PTC to the cervical lymph nodes. However, histopathological examination of lymph node A returned a negative result.

**Figure 3:**
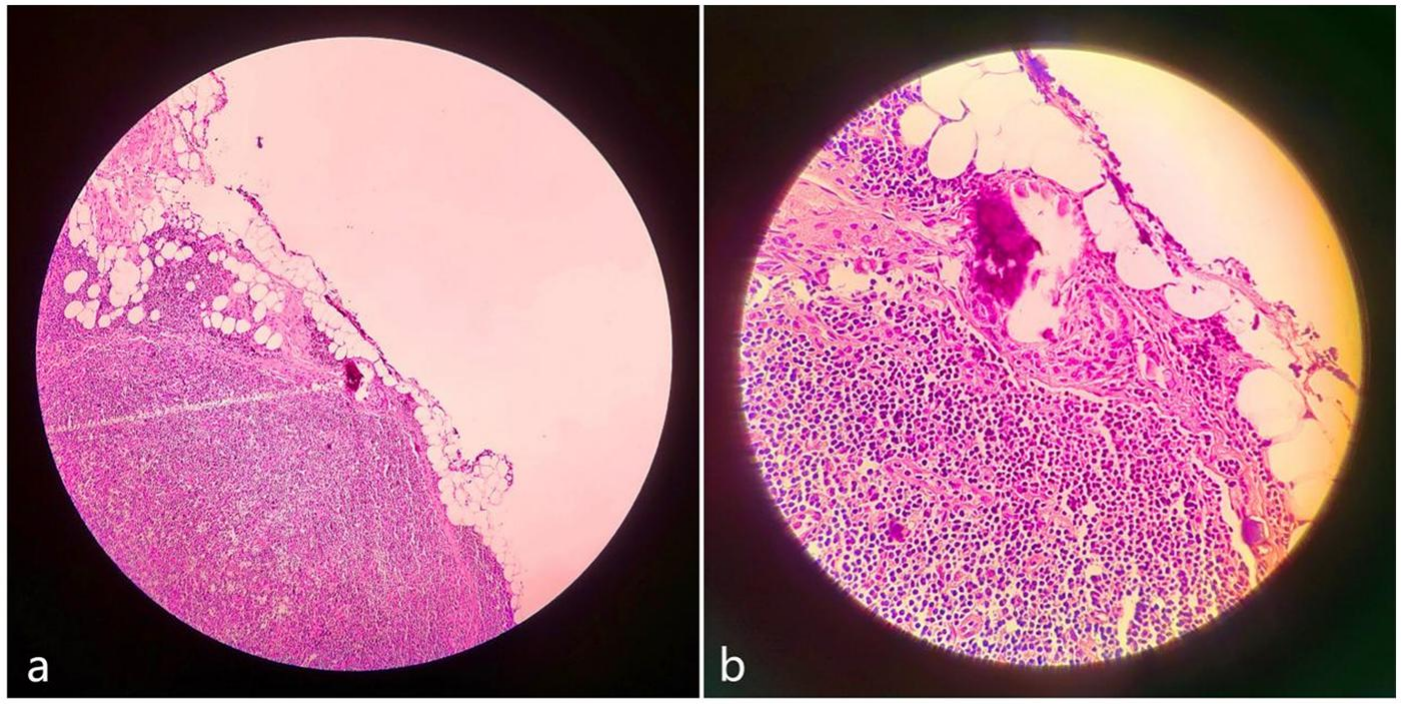
**a** the histopathological of lymph node A (HE ×100), **b** the histopathological of lymph node A (HE ×400).

We submitted the results to the pathologist for re-evaluation and requested a re-examination of the pathological tissue sections. After independent diagnoses by three pathology experts, the results were collectively analyzed. Based on comprehensive assessment, they unanimously confirmed the presence of micrometastases of PTC (diameter <2 mm) in lymph node A (Figure 3). Immunohistochemical results: HBME1 (-), CK19 (+), CD56 (+), galectin-3 (+).

## Follow-Up

Follow-up plan after discharge based on the 2025 American Thyroid Association management guidelines for adult patients with differentiated thyroid cancer (8). Individualization of decisions to initiate TSH suppression to below the reference range based on potential benefits and risks. We using supra-physiological doses of levothyroxine (LT4) to maintain thyroid-stimulating hormone levels (TSH) between 0.1 and 0.5 mU/L in patients with PTC to decrease the risk of recurrence or reduce the rate of disease progression. After 10 weeks of treatment, evaluation of the patient's neck ultrasound and serum thyroglobulin/anti-thyroglobulin antibody (Tg/TgAb) revealed no significant abnormalities; therefore, the current treatment regimen was continued. After 12 months of initial treatment, the patients underwent neck ultrasound and serum Tg/TgAb test, which revealed no significant abnormalities. Based on these findings, the doses of LT4 was adjusted to maintain TSH levels within the range of 0.5–2.0 mIU/L for subsequent management. Consequently, patient satisfaction was rated as “very satisfied”.

## Discussion

Papillary thyroid carcinoma (PTC) is a malignant tumor arising from follicular epithelial cells, accounting for approximately 95% of all thyroid cancer (9). Cervical LNM has been identified in approximately 30%–60% of patients with PTC through pathological examination (10). Usually, LNM occurs first in the central region (cLNM), followed by the lateral region (LLNM), which is an important indicator for prognosis assessment, surgical strategy formulation, and treatment option selection in patients with PTC. Also, it is a key risk factor that affects the recurrence and survival rates of patients (11).

Tg is a thyroid-specific glycoprotein synthesized and secreted by thyroid follicular epithelial cells. After binding to thyroxine, it is stored in the thyroid follicular lumen as a gelatinous complex. When lysosomes hydrolyze the bound Tg, thyroxine is released into the peripheral circulation, and a small amount of Tg is released into the bloodstream at the same time (12). High concentrations of thyroglobulin (Tg) were detected in fine-needle aspiration samples from cervical lymph nodes, indicating the presence of metastatic tumor cells originating from thyroid follicular epithelium (13). In this investigation, when the concentration of FNA-Tg exceeds that in peripheral serum, this finding suggests that lymph node A constitutes a cervical metastatic lymph node from PTC. Nevertheless, considerable debate persists regarding the optimal diagnostic cutoff for FNAB-Tg, along with uncertainties regarding whether serum thyroid-stimulating hormone (TSH), thyroglobulin antibody (TgAb), serum Tg, and thyroid tissue compromise the diagnostic accuracy of FNAB-Tg (14, 15).

The BRAF V600E mutation represents a critical oncogenic driver mutation in PTC. Accumulating evidence indicates that the prevalence of this mutation is significantly lower in benign thyroid nodules, follicular thyroid neoplasms, and medullary thyroid carcinoma. Leveraging this distinct mutational landscape, BRAF V600E mutation detection has been established as a validated auxiliary diagnostic biomarker for PTC in both clinical and research contexts (16). However, research directly investigating the pathological characteristics of LNM by detecting BRAF V600E mutation status in suspicious cervical lymph nodes of patients with PTC remains relatively limited. Studies reporting a detection rate of 78% in lymph node-positive cases, which introduced the possibility of BRAF 600E mutation assessment in predicting cervical LNM (17). Molecular analysis conducted in this study revealed a BRAF V600E mutation located in exon 15 in lymph node sample A. Correlative histopathological examination subsequently verified that the lesion represented a metastatic deposit of PTC within cervical lymph nodes. The study may provide a new clinical perspective for the management of lymph node abnormalities in PTC patients during preoperative or postoperative follow-up. Despite this, more cases should be enrolled, including not only those central lymph nodes, and more importantly, lateral cervical lymph nodes. Additionally, the cost reduction of BRAF V600E testing may promote its widespread application.

## Conclusion

The combination of FNA-Tg and BRAF V600E detection shows significant predictive efficacy for predicting cervical lymph node metastasis in PTC patients and plays an important guiding role in clinical practice and postoperative staging of patients. Despite this, more extensive clinical trials are still needed to provide new treatment strategies for the abnormally enlarged lymph nodes of PTC patients.

## Data Availability

The original contributions presented in the study are included in the article/Supplementary Material, further inquiries can be directed to the corresponding author.
